# Compound heterozygous variations in *IARS1* cause recurrent liver failure and growth retardation in a Chinese patient: a case report

**DOI:** 10.1186/s12887-022-03371-6

**Published:** 2022-06-07

**Authors:** Ting-Ting Zou, Hua-Qin Sun, Yu Zhu, Tian-Tian He, Wen-Wu Ling, Hong-Mei Zhu, Zi-Yuan Lin, Yan-Yan Liu, Shan-Ling Liu, He Wang, Xue-Mei Zhang

**Affiliations:** 1 Department of Pediatric Infectious Diseases, West China Second University Hospital, Sichuan University, Chengdu, 610041 China; 2 Key Laboratory of Birth Defects and Related Diseases of Women and Children (Sichuan University), Ministry of Education, West China Second University Hospital, Sichuan University, Chengdu, 610041 China; 3 SCU-CUHK Joint Laboratory for Reproductive Medicine, West China Second University Hospital, Sichuan University, Chengdu, 610041 China; 4 Department of Medical Genetics & Prenatal Diagnosis Center, West China Second University Hospital, Sichuan University, No.20, South Section 3, Renmin Road, Chengdu, Sichuan China; 5 Department of Ultrasound, West China University Hospital, Sichuan University, Chengdu, 610041 China

**Keywords:** Variations, *IARS1*, Recurrent liver failure, Growth retardation, Case report

## Abstract

**Background:**

Aminoacyl-tRNA synthetases (ARSs) are enzymes responsible for attaching amino acids to tRNA, which enables protein synthesis. Mutations in isoleucyl-tRNA synthetase (*IARS1*) have recently been reported to be a genetic cause for growth retardation, intellectual disability, muscular hypotonia, and infantile hepatopathy (GRIDHH).

**Case presentation:**

In this study, we reported an additional case of compound heterozygous missense variations c.701 T > C (p.L234P) and c.1555C > T (p.R519C) in *IARS1,* which were identified using medical exome sequencing; c.701 T > C (p.L234P) was a novel variant, and c.1555C > T (p.R519C) was found in GnomAD. Unlike other reported patients, this individual presented prominently with recurrent liver failure, which led to her death at an early age of 19 months. She also had significant growth retardation, muscular hypotonia, chubby and flabby face, recurrent loose stools, and abnormal brain computed tomography (CT), while zinc deficiency and hearing loss were not present. Studies in zebrafish embryo modeling recapitulated some of the key phenotypic traits in embryo development, neurodevelopment, liver development, and myogenesis, demonstrating that these variations caused a loss of gene function in *IARS1*.

**Conclusions:**

We have found a novel mutation point c.701 T > C (p.L234P) in *IARS1*. Compound heterozygous mutations of c.701 T > C (p.L234P) and c.1555C > T (p.R519C) in *IARS1* are pathogenic, which can cause GRIDHH in child.

## Background

Aminoacyl-tRNA synthetases (ARSs) are enzymes responsible for attaching the correct amino acid to tRNA, which allows protein synthesis; tRNA synthetase deficiencies are a growing group of genetic diseases associated with tissue-specific, mostly neurological, phenotypes [1, 2]. *IARS1* is located on chromosome 9q22.31 and consists of 34 exons. Mutations in isoleucyl-tRNA synthetase (IARS1 OMIM # 600709) have been linked to growth retardation, intellectual disability, muscular hypotonia, and infantile hepatopathy (GRIDHH, OMIM 617093), which was first reported by Kopajtich et al. in 2016 [3]. It is a rare autosomal recessive multisystem disorder, with major phenotypic characteristics including overall growth retardation in prenatal and postnatal, impaired intellectual development, hypotonia, and variable hepatopathy. Other individual features including recurrent infections, zinc deficiency, diabetes mellitus, hydronephrosis, esophagitis were also reported in previous studies. The treatment was mainly based on symptomatic and supportive therapy like antibiotics for infections, zinc supplementation for zinc deficiency, insulin for diabetes mellitus, fresh frozen plasma and vitamin K for coagulopathy. To date, there have only been some reports on patients with variable-severity phenotypes [2–7].

Here, we described total clinical characteristics of the case of a Chinese patient with compound heterozygous variations in *IARS1*. Studies in zebrafish embryo models showed the pathogenicity of these two variations.

## Case presentation

### Clinical presentation

The proband was the first baby of non-consanguineous Chinese parents. The parents were healthy without any notable medical conditions, and there was no known history of familial liver or neurological disorders. The pregnancy was complicated by threatened miscarriage in the early stage and abnormal second-trimester screening with a high risk of 1/16 for Down syndrome, but amniocentesis revealed a normal karyotype of 46, XX. Routine ultrasound examination during the second trimester was normal, but during the third trimester (about 30 gestational weeks), there was remarkable growth delay and oligohydramnios. She was delivered via spontaneous vaginal delivery at 37 weeks of gestation, plus 4 days. The birth weight was 2100 g (− 2.79SD). She was admitted to a local hospital half an hour after birth for low birth weight. During her hospitalization, the total bilirubin, composed of 91–100% unconjugated bilirubin, increased from 60.6 μmol/L (normal range: 3–22 μmol/L) at 6 hours up to 215.1 μmol/L at 87 hours. Liver function test also revealed increased aspartate aminotransferase (AST) (164 U/L, normal range: 0–40 U/L), gamma-glutamyl transpeptidase (GGT) 243 U/L (normal range: 0–43 U/L), and decreased cholinesterase (2294 U/L, normal range: 5000–12,000 U/L). The IgM of toxoplasma, rubella virus, herpes simplex virus, and cytomegalovirus (CMV) were negative. The hepatitis virus B and C were negative. She did not undergo ultrasound examination of liver this time. She was discharged about 1 week later and the diagnoses were low birth weight at term and neonatal jaundice. Her newborn screening metabolic tests yielded normal results. Thus, further genetic consultation did not recommend. The jaundice faded within 2 weeks and the girl had a poor appetite during the whole infantile period according to the parents.

Postnatal growth was also retarded, and her weight was 2.5 kg (− 3.34SD), 6.5 kg (− 2.25SD), and 7.5 kg (− 2.78SD) at one, nine, and 19-month-old, respectively. The psychomotor development was delayed; she could not turn over until 5-month-old, sit until 8-month-old, and stand up without aid until 19-month-old. She began to babble at 5-month-old and could not say “baba” or “mama” at 19-month-old. She had loose stools recurrently from 3-month-old, but without bloody stools.

**Fig. 1:**
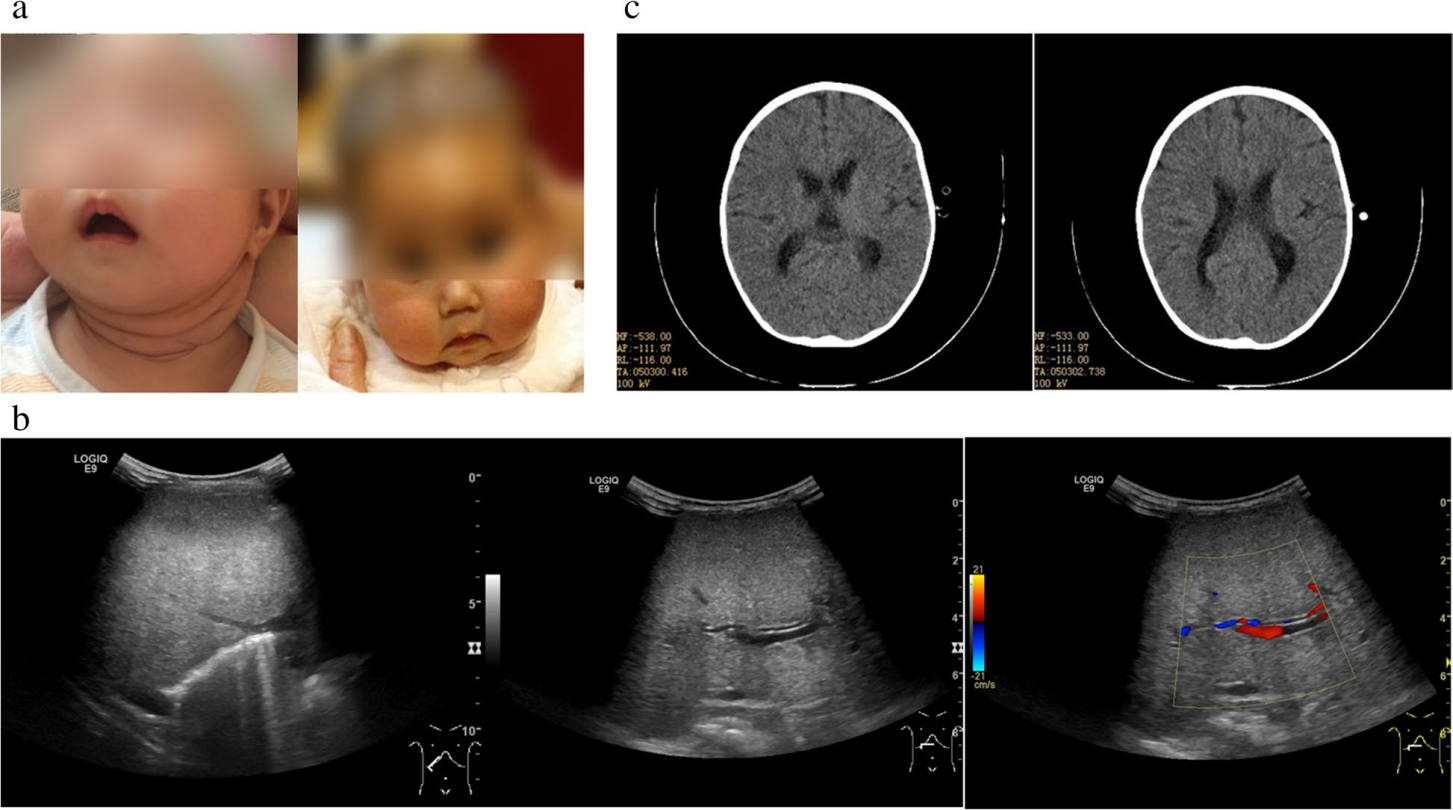
Clinical manifestations. **a** Face feature. This girl had abnormal fat, round face, and soft translucent skin. **b** Ultrasound examination. Conventional B-mode ultrasound (left) showed that the liver was significantly enlarged and the echo of the liver parenchyma was coarse, suggesting the possibility of cirrhosis. The B-mode ultrasound (middle) and color Doppler flow imaging (CDFI)(right) showed enlargement of hepatic artery (arrows). **c** CT scan of brain. The boundary between gray matter and white matter was not clear, the sulci and cistern were slightly widened and deepened, the left lateral ventricle was slightly widened, about 1.0 cm(arrows)

At 9-month-old, she was admitted to our hospital for acute gastroenteritis caused by astrovirus infection. On physical examination, the girl presented with obvious chubby and flabby face (Fig. 1a), and the skin was hyperelastic. She also had muscular hypotonia and joint laxity. A microcephaly was notable, with a head circumference of 40 cm (− 3.2SD). Abdominal examination demonstrated an enlarged liver with its lower edge 7 cm below the right costal margin, and an enlarged spleen with its lower edge 2.5 cm below the left costal margin. The textures of the liver and the spleen were median. Laboratory examination revealed severe liver and coagulation dysfunction. Vitamin D levels were slightly decreased (Table 1). The muscle enzyme, blood glucose, lactate level, and AFP levels were all normal. Hepatitis viruses, including hepatitis A, B, C, D, E, CMV, and EBV were all negative. The autoantibodies were negative. Ultrasound examination of the liver revealed hepatomegaly and hepatic cirrhosis, with compensatory enlargement of the hepatic artery (Fig. 1b). With liver failure triggered by infections and growth retardation, progressive deterioration of liver function, and muscle involvement, inherited metabolic liver disease was considered. The genetic test for medical exome sequencing was sent at 9 months and 22 days. The patient was given symptomatic and supportive treatment, including transfusion of fresh frozen plasma and prothrombin complex, albumin and fibrinogen, supplements of vitamin A, D, E, and K, and infusion of glutathione and S-adenosylmethionine. Her liver function recovered and was sustained for a period of time with supportive treatment. However, when she was 19-month-old, she developed severe pneumonia, and the liver function collapsed again (the biochemical changes are shown in Table 1). The blood zinc level was tested, with normal results. The CT scan of the brain indicated that the boundary between the gray matter and white matter was not clear, the sulci and cistern were slightly widened and deepened, and the left lateral ventricle was slightly widened, approximately 1.0 cm (Fig. 1c). Oxygen inhalation through nasal catheter was needed when she was admitted. Anti-infection and supportive treatments were no longer effective this time. The mechanical ventilation was needed at the final stage. The patient died of liver failure and severe infection at last. The clinical and genetic findings of this patient are summarized in Table 2.

**Table 1:** Biochemical change of the patient in different ages

Biochemical item	9 months old	19 months old	Normal range
ALT	745	231	<49U/L
AST	4372	1172	<40U/L
TBIL	36.9	23.8	5~21μmol/L
DBIL	31.6	21.8	<6.8μmol/L
TBA	254.8	254.8	0~10μmol/L
ALB	21.0	24.8	38.0~54.0g/L
PT	29.6	25.2	8.7~14.7sec
APTT	123.6	212.0	17.5~37.5 sec
INR	2.47	2.09	0.8~1.5
Fg	102	<50	200~400mg/dl
VitD^a^	23.4	21.90	30~100ng/ml
Zn^a^	-	54.62	43.55~95.63μmol/L
NH_3_	40.0	76.0	9~30μmol/L

*Abbreviation*: *ALT* Alanine aminotransferase, *AST* Aspartate Aminotransferase, *TBIL* Total bilirubin, *DBIL* Direct bilirubin, *TBA* Total bile acid, *ALB* Albumin, *PT* Prothrombin time, *APTT* Activated partial thromboplastin time, *INR* International Normalized Ratio, *Fg* Fibrogen, *VitD* Vitamin D, *Zn* Zinc

^a^The patient received Vitamin AD drops and Calcium Zinc Gluconate Oral Solution daily based on the advice of pediatrician of child health care departement

**Table 2:** Clinical and genetic findings of patients with IARS1 mutations

No.	Ethnic	Sex	ALV	AO	IARS1 mutations	Clinical features				
					cDNA;protein	Growth Retardation	Intellectual Disability	Muscular Hypotonia	Infantile Hepatopathy	Other features
P1 [3]	German	M	18.7y	Prenatal (IUGR)	c.[1252C>T], [3521T>A]; p.[Arg418^a^],[lle117Asn]	+	+	+	+	zinc deficiency, recurrent infections, esophagitis, microcephaly.
P2 [3]	Japanese	F	19y	Prenatal (IUGR)	c.[760C>T], [1310C>T]; p.[Arg254^a^], [Pro437Leu]	+	+	⁃	+	zinc deficiency, diabetes mellitus, sensoneurinal hearing loss, epilepsy.
P3 [3]	Austrian	M	3y	Prenatal (IUGR)	c.[1109T>G], [2974A>G]; p.[Val370Gly],[Asn992Asp]	+	+	+	+	zinc deficiency, recurrent infections, recurrent liver crises triggered by infections, chubby cheeks, microcephaly.
P4 [4]	Israeli Arab	M	4y	Prenatal (IUGR)	c.[2215C>T], [1667T>C]; p.[Arg739Cys],[Phe556Ser]	+	+	⁃	+	zinc normal, hydronephrosis, joint hyperlaxity, hyperelastic, doughy-like and transparent skin, microcephaly.
P5 [5]	Polish	M	7y	Prenatal (IUGR)	c.[2011delC], [206C>T]; p.[Gln671fs],[Thr69lle]	+	+	+	+	zinc deficiency, recurrent infections, drumstick fingers
P6^a^	Chinese	F	1.58y	Prenatal (IUGR)	c.[701T>C], c.[1555C>T]; p.[L234P], p.[R519C]	+	+	+	+	zinc normal, recurrent infections, recurrent liver failure triggered by infections, microcephaly, chubby cheeks, hyperelastic skin, vitamin D deficiency

*Abbreviation*: *M* Male, F female, *ALV* Age at last visit, *AO* Age of onset, *IUGR* Intrauterine growth retardation. ^a^P6 is the present patient

### Identification of two missense variants

Medical exome sequencing was performed on the proband’s DNA sample at Beijing Mygenostics Inc. (Beijing, P.R. China) using the GenCap Exome Capture Kit (MyGenostics, Beijing, China) on the Illumina NextSeq 500 (Illumina, San Diego, CA, USA). The mean depth of coverage of the sequenced sample was 200×. Sequencing data were aligned to the human reference genome using BWA software (http://bio-bwa.sourceforge.net/). Variations were identified using GATK software (https://software.broadinstitute.org/gatk/). Detected variants were annotated and filtered using ANNOVAR software (http://annovar.openbioinformatics.org/en/latest/), and associated with multiple databases, such as 1000 genome, ESP6500, dbSNP, GenomAD, Inhouse (MyGenostics), HGMD. Pathogenicity was predicted using SIFT, PolyPhen-2, MutationTaster, and GERP. The detected variations were confirmed by bidirectional Sanger sequencing on an ABI 3130 Genetic Analyzer (Applied Biosystems, Waltham, MA, USA) and verified in the parents.

**Fig. 2:**
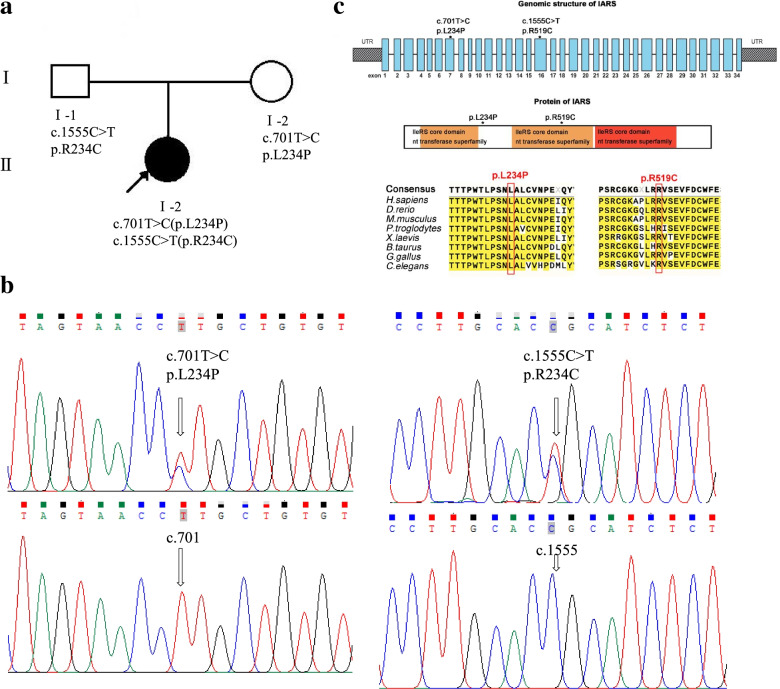
*IARS1* variations and gene structure. **a** Pedigrees of this family with recessive inherited mutations in *IARS1*. This patient had two missense variants, and her parents each carried one of the mutations. **b** Sanger sequencing for these two variations in this family. Left panel is variant c.701 T > C (p.Leu234Pro) (upper) and the normal control at this site (lower); right panel is variant c.1555C > T (p.R519C) (upper) and the normal control at this site (lower). **c** Structure of *IARS1* (NM_002161, NP_002152) with known conserved protein domains in the gene product and localization and conservation of amino acid residues affected by variants identified in this family. Intronic regions are not drawn to scale

We identified two missense variants, c.701 T > C (p.Leu234Pro) (Hg19 chr9–95,043,072) and c.1555C > T (p.Arg519Cys) (Hg19 chr9–95,027,355), in the *IARS1* gene (NM_002161, NP_002152). These results were confirmed using Sanger sequencing, and showed that the parents each carried one of the variants; c.701 T > C (p.L234P) in the mother, and c.1555C > T (p.Arg519Cys) in the father (Fig. 2a and b). The c.701 T > C variant is located in exon 7, which is close to the IleRS core domain, resulting in a Leu234Pro substitution. The other variation c.1555C > T is located in exon 16 of the IleRS core domain, resulting in an Arg519Cys substitution (Fig. 2c). These two missense variations change evolutionarily highly conserved amino acid residues (Fig. 2c). The c.701 T > C(p.L234P) variant had not been previously reported, while the variants c.1555C > T(p.Arg519Cys) was found in GnomAD (rs147238476). Both were predicted to be probably damaging and deleterious using the protein function prediction software Polyphen2, SIFT, and Mutation Taster. There were no other plausible candidate variants consistent with the patient’s clinical phenotype and recessive (autosomal or X-linked) inheritance.

Blood samples were obtained from the patient and her parents, and genomic DNA was isolated from peripheral blood according to manufacturer instructions (CWBIO, Beijing).

### R519C and L234P variants lead to loss of function of *IARS1* gene in zebrafish model

**Fig. 3:**
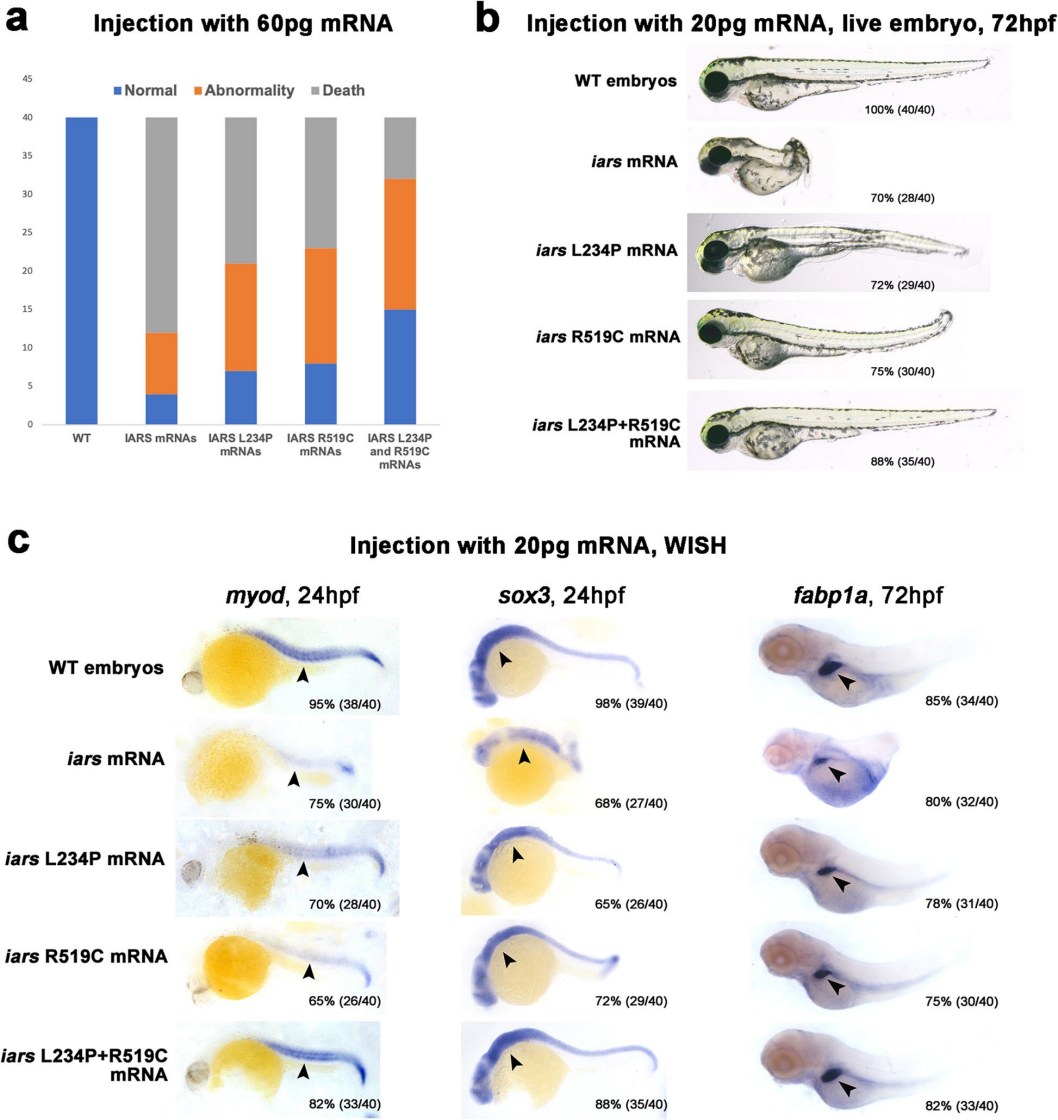
R519C and L234P variants lead to loss of function of the *IARS1* gene in the zebrafish model. **a** High doses of IARS1 and its variant mRNAs (60 pg) produced a high rate of embryonic death and severe embryonic deformities. **b**, **c** IARS1 R519C and L234P caused a loss of gene function of *IARS1* during zebrafish embryogenesis. *IARS1* variations in mRNA failed to impair embryo development (**b**), myogenesis, neurodevelopment, and liver development (**c**). Live embryos and fobp1a expression at 3 dpf; sox3 and myod expression at 24 hpf stage, all embryos shown are lateral views anterior to the left. The percentages and numbers indicated in each picture are the ratio of the number (left in bracket) of affected embryos with phenotypes similar to what is shown in the picture and the total number (right in bracket) of observed embryos

These two variants change evolutionarily highly conserved amino acid residues in more than one species (Fig. 2c). To determine whether the *IARS1* R519C and L234P mutations led to loss of function in vivo, we constructed a human *IARS1* gene and its mutation using the pcDNA3.1+ vector. After mRNA synthesis, embryos were injected. When zebrafish embryos were injected with high doses of mRNAs (~ 60 pg), *IARS1* mRNAs produced a high rate of embryo death at 24 h post fertilization (hpf), whereas the mutant mRNAs induced severe embryonic deformities (Fig. 3a). However, when we decreased the mRNA injection doses to ~ 20 pg, embryos injected with *IARS1* mRNAs showed an obverse phenotype of shorter body axis in 70% of embryos, but mutant mRNA injection did not generate severe embryonic deformities. Especially, injection of *IARS1* R519C and L234P double mutation mRNAs just induced abnormality in 12% of embryos, and 88% of embryos showed normal phenotype (Fig. 3b). These results suggested that the *IARS1* R519C and L234P mutations in the human *IARS1* gene caused loss of function.

Based on the clinical multisystem symptoms of this patient, we investigated liver, brain, and muscle development during embryogenesis using whole-mount in situ hybridization (WISH) for marker analyses. Results showed that the staining of myogenic marker myod, central neuron system marker sox3, and hepatocyte marker fabp1a decreased significantly in embryos injected with ~ 20 pg *IARS1* mRNAs. In detail, 75% of embryos showed decreased expression of myod when injected with *IARS1* mRNAs, 68% of embryos showed decreased expression of sox3, and 80% of embryos showed decreased expression of fabp1a. Meanwhile, embryos injected with the same doses of single *IARS1* mutation mRNAs (R519C or L234P) showed weak staining of the three detected marker genes. Importantly, injection with the same doses of *IARS1* R519C and L234P double mutation mRNAs showed little effect on the staining of these marker genes. Detailly, just 18% of embryos (82% normal) showed decreased expression of myod when injected with double mutation mRNAs, 12% of embryos (88% normal) showed decreased expression of sox3, and 18% of embryos (82% normal) showed decreased expression of fabp1a (Fig. 3c). Taken together, these results demonstrated that *IARS1* R519C and L234P mutations caused a loss of gene function in *IARS1*.

All animals were handled in accordance with the Guide for the Care and Use of Laboratory Animals, and the study was approved by the Institutional Animal Care and Use Committee of Sichuan University. The human *IARS1* coding region sequence was obtained from Dharmacon (Catalog Number OHS5893–202499238, Lafayette, CO, USA). Zebrafish embryo microinjection, whole-mount in situ hybridization, grayscale measurement, point mutation expression plasmid construction, and statistical analyses were performed as previously described [8].

## Discussion and conclusions

Mutations in isoleucyl-tRNA synthetase (*IARS1* OMIM # 600709) have been linked to GRIDHH (OMIM 617093) with an autosomal recessive inheritance pattern in five patients [3–6] (Table 2), described as a rare multisystem disorder reported first by Kopajtich et al. in 2016. Another case was reported in a patient who was suspected to have mitochondrial disease, along with the phenotype of GRIDHH, including growth retardation, hepatopathy, muscle weakness, and failure to thrive [7].

Our study supported the association between recessive mutations in the *IARS1* gene and the clinical characteristics of GRIDHH. However, there were some variations in the clinical phenotype in this subject. Unlike other patients who could live longer until teenage years, this patient had severe and recurrent liver failure triggered by recurrent infections, and these prominent problems led to her early death at 19-month-old. She also had significant overall growth retardation with prenatal onset, impaired intellectual development, muscular hypotonia, and chubby and flabby face, similar to most previously reported patients. However, several other individual features were distinct in this case, including oligohydramnios and hepatosplenomegaly which were only seen in an Israeli Arab boy and one Polish boy, respectively [4, 5]. Interestingly, loose stools were present in this girl at an early age (3-month-old), which was just reported in a recent case [6]. The patient also had obvious abnormalities on CT with no clear boundary between the gray matter and white matter, the sulci and cistern were slightly widened and deepened, and mild left ventriculomegaly, which has seldom been reported.

Low zinc levels have been reported in some previous cases as a significant clinical feature. However, the zinc level was normal in this case, although the patient had severe liver dysfunction. In Orenstein’s case [4], the zinc level was normal and the boy also had severe liver dysfunction but did not have recurrent infections, whereas in Fagbemi’s case [6], both plasma zinc concentration and liver function were normal. In a study by Kopajtich [3], zinc supplementation had no direct effect on *IARS1* activity; hence, reduced zinc levels were not directly related to *IARS1* mutations. Based on this information, it is likely that low zinc levels may not be the phenotype of *IARS1,* but a secondary manifestation.

In the case presented in this study, both variants were missense mutations. One was close to the IleRS core domain, and the other was in the IleRS core domain, and both variants change evolutionarily highly conserved amino acid residues in more than one species. To evaluate the functional consequences of these *IARS1* variants identified in this patient, morphological studies in zebrafish were performed. When zebrafish embryos were injected with high doses of mRNAs, they showed delay in embryonic development with shorter body axis, and multisystem markers demonstrated that the two mutations R519C and L234P caused a loss of gene function of *IARS1*, which successfully revealed the functional consequences of disease-associated for these two novel *IARS1* mutations. Meanwhile, WISH studies for multisystem development during embryogenesis with hepatocyte marker fabp1a, central neuron system marker sox3, and myogenic marker myod, indicated *IARS1* R519C and L234P mutations caused a loss of gene function of *IARS1,* which resulted in multisystem disfunction in line with the clinical observations in this patient.

In conclusion, GRIDHH is a rare genetic disorder caused by *IARS1* mutations in autosomal recessive. We have found a novel mutation point c.701 T > C (p.L234P) in *IARS1*. Compound heterozygous mutations of c.701 T > C (p.L234P) and c.1555C > T (p.R519C) in *IARS1* are pathogenic, which can cause GRIDHH in child. Our study provided further insights into the new evidence on the phenotype and genotype spectrum of *IARS1* mutations.

## Data Availability

Not applicable.

## Abbreviations

ARSs: Aminoacyl-tRNA synthetases
IARS1: Isoleucyl-tRNA synthetase
GRIDHH: Growth retardation, intellectual disability, muscular hypotonia, and infantile hepatopathy
GnomAD: Genome Aggregation Database
CT: Computed tomography
OMIM: Online Mendelian Inheritance in Man
GATK: The Genome Analysis Toolkit
HGMD: Human Gene Mutation Database
SD: Standard Deviation
AST: Aspartate aminotransferase
GGT: Gamma-glutamyl transpeptidase
IgM: Immunoglobulin M
AFP: Alpha-fetoprotein
CMV: Cytomegalovirus
EBV: Epstein-Barr virus
WISH: Whole-mount in situ hybridization
